# Etymologia: Cytokines

**DOI:** 10.3201/eid2407.ET2407

**Published:** 2018-07

**Authors:** Thomas J. Gryczan

**Keywords:** etymologia, cytokines, cell signaling, immune cells, immunomodulation, lymphocytes, interferons, interleukins

## Cytokines [si′to-kīnes]

From the Greek *cyto* (cavity or cell) and *kine* (movement), cytokines are proteins involved in cell signaling and function as immunomodulating agents. Cytokines are produced by immune cells (e.g., macrophages, B and T lymphocytes, mast cells, neutrophils, natural killer cells), endothelial cells, fibroblasts, and stromal cells.

Although the term cytokine had not yet even been defined, interferon-α, the first cytokine known, was identified in 1957 as a protein that interfered with virus replication. Activities of interferon-γ and interleukin-2 were identified in 1965. Macrophage migratory inhibitory factor was identified in 1966. In 1969, Dumonde and colleagues proposed the term lymphokine to describe proteins secreted from lymphocytes. Proteins derived from macrophages and monocytes were later called monokines. In 1974, Cohen and colleagues reported production of macrophage migration inhibitory factors in virus-infected fibroblasts, which led (finally) to proposal of the term cytokine. 
